# Adapting the WHO BeSD COVID-19 Survey to Examine Behavioral and Social Drivers of Vaccine Uptake Among Transgender, Intersex, and Disability Communities in India

**DOI:** 10.3390/vaccines13111095

**Published:** 2025-10-24

**Authors:** Eesha Lavalekar, Sharin D’souza, Harikeerthan Raghuram, Namdeo Dongare, Mohammed A. Khan, Chaitanya Likhite, Gauri Mahajan, Pabitra Chowdhury, Aqsa Shaikh, Sunita Sheel Bandewar, Satendra Singh, Anant Bhan

**Affiliations:** 1 Initiative for Health Equity, Advocacy and Research (iHEAR), Sangath, Bhopal 462042, India; eesha@cmhlp.org (E.L.);; 2 Youth Voices Count, Iloilo City 5000, Philippines; 3 Department of Community Medicine, Hamdard Institute of Medical Sciences and Research, New Delhi 110062, India; 4 Forum for Medical Ethics Society, Mumbai 400025, India; 5 Department of Physiology, University College of Medical Sciences, Delhi 110095, India

**Keywords:** COVID-19 vaccination, vaccine equity, transgender, disability, intersex, community-based participatory research, BeSD framework

## Abstract

Background: During the COVID-19 pandemic, transgender and gender-diverse (TGD) people and people with disabilities in India faced disproportionate barriers to accessing vaccination services. Building on previous studies, this study explored the experiences of COVID-19 vaccine access in these two marginalized communities, using the WHO Behavioral and Social Drivers (BeSD) framework. Methods: Keeping community-based participatory methods (CBPR) at heart, we conducted a survey adapted from the BeSD COVID-19 survey tool. The survey was adapted using insights from a prior study, a literature review, stakeholder consultations, and discussions with a community leadership group (CLG) and an advisory board (AdB). Participants were recruited through transgender, gender-diverse, and disability rights networks. Data were analyzed descriptively, using percent analysis, and psychometrically, using exploratory factor analysis on polychoric correlations. Results: The adapted BeSD survey tool showed a high 0.85 (*p* < 0.05) internal consistency and criterion validity. Moreover, it showed a high willingness to be vaccinated (for ease of access to other services and community responsibility); however, systemic barriers hindered vaccination access. TGD people and people with disabilities faced multiple barriers in being vaccinated. The TGD community reported documentation mismatches and mistrust in health systems. People with disabilities reported mobility challenges, escort dependence, financial challenges, and variable accessibility at vaccination sites. Both groups faced digital exclusion, received inadequate information that did not address their specific needs, and experienced inconsistent implementation of inclusive policies. Community-led facilitation led to more uptake. Conclusions: Vaccine willingness alone is insufficient to ensure that vaccines reach everyone. Addressing trust deficits, infrastructural barriers, and digital exclusions requires diligent attention and commitment from the government to mitigate broader challenges faced by TGD people and people with disabilities.

## 1. Introduction

The development of COVID-19 vaccines has been one of the most urgent and powerful scientific achievements of the 21st century, and vaccination has been a key strategy to reduce the spread of SARS-CoV-2 [1,2]. However, research has also shown that people from different marginalized communities, like racial and ethnic minorities, LGBTQIA+ people, people with disabilities, people living with HIV, and people experiencing homelessness, can experience structural, community, and individual-level barriers to vaccination, which can lead to under-vaccination in these communities [3].

In 2021, India rolled out the world’s largest national COVID-19 vaccination program; however, data from the same year also shows that vaccination coverage among transgender and disability communities in India was much lower when compared to the general population [4,5]. In fact, the lack of reliable disaggregated data for both communities has also been a barrier to accountability. For instance, the Indian government provided contradictory data for vaccination coverage rates among people with disabilities on two different occasions [6]. Further, on the vaccination appointment booking portal, the unclear category of “Others” was provided instead of “Transgender” [7]. Moreover, previous research shows that vaccination systems can be ableist and trans exclusionary [7], and that community engagement can help build trust, address anxieties, and bring vaccinations to the two communities [8].

Barriers to vaccination for individuals in the two communities began with concerns around how the COVID-19 vaccines might interact with their specific health needs, like specific disabilities, or hormone therapy and gender affirming surgeries for transgender people [9,10]. Additionally, medical and institutional mistrust and fear of violence and mistreatment, especially among trans and gender-diverse communities, presented structural barriers to vaccine access [3]. Research also shows that people with disabilities generally have a more positive attitude towards vaccinations [11].

The journey from deciding to be vaccinated to receiving the vaccination also involved additional hurdles. In India, digitally mediated appointment booking for vaccination through the COVID-19 Vaccine Intelligence Network (CoWIN) was inaccessible to people with disabilities, but also to many others who found booking slots challenging due to limited digital literacy and poor connectivity [12,13]. Moreover, identity verification through legal documents such as Aadhaar was required to be able to receive the vaccine. This was a challenge for trans and gender-diverse individuals whose official documents did not align with their gender identity, or those who did not have access to their documents [14]. Later, the government developed home-vaccination protocols, specific guidelines for people with disabilities and trans people, and protocols for being vaccinated without prescribed identity documents [15,16].

A cross-sectional study from South Korea also illustrated that individuals with disabilities were less likely to receive COVID-19 vaccinations than those without disabilities, despite being more vulnerable to COVID-19-related mortality [17]. For people with disabilities, socioeconomic challenges, built environment inaccessibility, challenges in accessing home vaccination, limited transportation facilities, and lack of trained healthcare personnel became barriers to access [18]. Due to a lack of accessible infrastructure and resources, reports of Deaf individuals struggling to access sign language support or people with visual impairments being unable to navigate crowded registration sites highlighted gaps in inclusive planning [19].

In the TGD community, social stigma, discrimination, lack of access, and non-prioritization in vaccine drives were identified as factors affecting vaccine acceptance [20]. Further, economic and social marginalization, lack of transportation, lack of insurance coverage, loss of employment, and housing insecurity also shaped vaccine attitudes in the TGD community [3]. Further infrastructure at many vaccine centers was binary, with bathrooms and queues for only men and women [7]. In India, there were later directives for vaccine centers to create special booths/counters for trans people and people with disabilities to address the disparities in vaccination rates [21,22].

Furthermore, there is limited acknowledgement of the role of compounded vulnerabilities in health access. It is increasingly critical to understand how overlapping vulnerabilities can deepen systemic exclusion and reduce the likelihood of equitable vaccine uptake. For example, a report from Pakistan showed that trans people living with HIV experienced a lot of fear and stigma about the COVID-19 vaccination, which slowed down the uptake [23]. Similarly, women with disabilities were shown to have a lower likelihood of COVID-19 vaccination [17].

Frameworks like the Behavioural and Social Drivers (BeSD) of Vaccination framework by the WHO examine vaccine uptake through four domains: confidence, motivation, social processes, and practical access. They are incredibly important to understand and address contextual challenges to vaccine uptake [24]. However, these frameworks often exclude the unique needs of people with disabilities and gender minorities, like a lack of information on specific health needs, fear of stigma, accessibility, etc. Thus, there is a need to expand such frameworks to include the needs of marginalized communities that are not adequately prioritized within vaccination systems despite having poor immune status linked to disabilities, or poorer health in general, and being more vulnerable to COVID-19 mortality [20,25]. Intersex people are another such community. Intersex people are born with “physical, hormonal or genetic features that are neither wholly female nor wholly male; or are a combination of female and male; or neither female nor male”. Intersex variations occur in approximately 1.7 per cent of all births [26]. Intersex people also have unique health needs, depending on their variations, and also experience non-consensual surgeries at a young age that can have life-long health impacts. While research talks about LGBTQIA+ people and their experiences with vaccination, a big gap exists in understanding intersex experiences and barriers specifically. To address this gap in vaccination frameworks, and the subsequent lack of disaggregated data on vaccine acceptance among TGD, intersex and disability communities in India, the present study aimed to adapt and apply the BeSD framework to understand COVID-19 vaccination experiences among intersex and TGD communities and people with disabilities in India.

## 2. Objective of the Study

This study had two key objectives. First, to adapt and validate the WHO Behavioural and Social Drivers (BeSD) tool for use among transgender, disability, and intersex (TGDI) populations in India, using a community-based participatory research process. Second, to explore descriptive information on COVID-19 vaccine uptake, including confidence, motivation, social influences, and access-related barriers, using an adapted WHO-BeSD survey for the transgender and disability communities. The survey could not be conducted with people with intersex variations, due to challenges in recruitment.

## 3. Methodology

### 3.1. Research Methods

**Figure 1:**
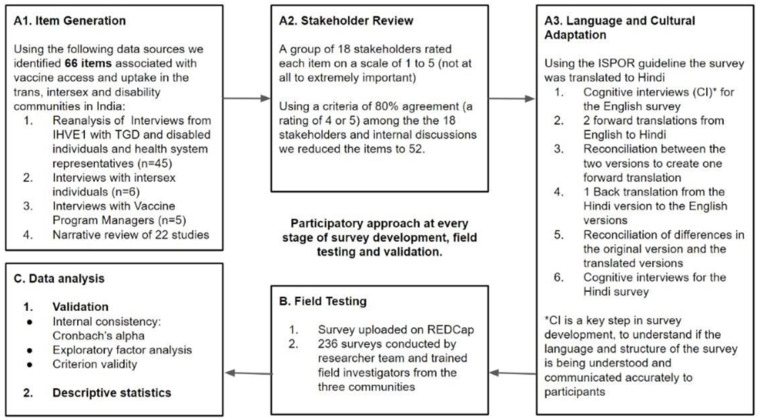
Flowchart showing different stages of the research process.

iHEAR VaccineEquity 2.0 was an exploratory sequential mixed method study guided by CBPR and the methodology, followed by the BeSD working group [24]. It followed three phases with multiple subcomponents, as illustrated in Figure 1.

The community-based participatory approach: Taking a participatory approach throughout the lifespan of the study, the research was co-led by community members from the TGD, intersex, and disability communities, as well as health systems and civil society representatives. They were present in the project team, a community leadership group (CLG), and an advisory board (AdB), and were recruited as consultants and reviewers. The CLG (lived experience and knowledge of field realities) and the AdB (subject and methodological expertise) shaped the project through their guidance on research design, survey design, recruitment, survey conduct, and study dissemination. The CLG consisted of 10 activists and advocates from TGD, intersex, and disability communities, including members of organizations like Intersex Human Rights India, Humsafar Trust, Cross the Hurdles, Sruti Disability Rights Center, and state-level collectives. The CLG and ADB convened five times to seek guidance and input at every stage, with one of these being an in-person meeting in Delhi at the study design stage, where we workshopped what the survey should look like. The AdB, on the other hand, comprised 5 individuals to represent expertise in vaccine equity, public health, social medicine, disability inclusion, and global health. The AdB convened 4 times to discuss the design and progress of the survey, mitigate challenges in recruitment, and provide input on dissemination approaches.

**A.1 and A.2. A tool development phase:**

Item generation and stakeholder review

We explored four data sources to generate an exhaustive list of 66 items influencing vaccine uptake in the three communities. Each of these items was reviewed by 18 stakeholders, who rated the items on a scale of 1 to 5 in order of increasing importance, with respect to their influence on vaccine uptake and access among the three communities. A rating of 4 or 5 was considered to be agreement for inclusion in the survey. If 80% or more of the stakeholders gave an item a score of 4 or 5, we considered the item for inclusion in the survey. After several rounds of internal discussion, we developed a survey with 52 items, retaining many questions from the original BeSD and adding community-specific questions for the three communities. The 4 data sources were as follows:

- Reanalysis of the data collected through 45 interviews conducted in a previous study by the research team between 2021 and 2023, which looked at structural barriers to vaccine access among the TGD and disability communities [7];
- A narrative review of the literature on vaccine access and uptake among the three communities in the Global South;
- Semi-structured interviews with intersex individuals from different states in India during the item generation phase;
- Semi-structured interviews with vaccine program managers from different governmental and non-governmental organizations across India.

Many items that we identified for the survey came up across data sources, like perceived risk to self and confidence in COVID-19 vaccine safety, while others came up only in one data source. For example, access to information on the health needs of intersex people emerged only in interviews with intersex individuals.

The interviews with program managers offered us insights into aspects such as survey design, the importance of using simple terminology, rigorous enumerator training, and gathering socio-demographic information to understand social determinants of vaccination among these communities. We could not include intersex community members in the survey due to challenges involved in their recruitment.

**A.3. Translation and Cultural Adaptation (Cognitive Interviews):**

After an initial survey was designed, to understand how it was being received, we conducted a total of nine cognitive interviews (a method used to evaluate survey questions by examining a participant’s thought process as they understand and respond to them), with four in English and five in Hindi, as Hindi is widely spoken in the selected states, whereas it was likely that English might not always be comfortable to everyone that we wanted to reach.

The insights gathered from the interviews provided valuable recommendations across multiple aspects of the survey, which informed changes in the final survey (See Table S1).

**B. A field-testing phase:**

Two key activities comprised the field-testing phase, namely

***B.1. Enumerator training:***

Once the expanded survey tool was finalized (with the help of cognitive interviews) with 52 items and 23 socio-demographic questions, 4 training sessions were conducted to train a total of 26 enumerators to collect data in four states, which were chosen based on available community networks and vaccination uptake: Maharashtra, Madhya Pradesh, Uttar Pradesh and New Delhi. These sessions focused on essential components of data collection, including survey administration, adherence to ethical standards, and strategies for effective participant engagement, particularly when addressing sensitive topics. Out of the 26 trained enumerators, 9 collected data (34.6%). A total of 4 enumerators were from the TGD community, and 5 enumerators were from the disability community. The feedback from the enumerators was very positive, with many commending the practical exercises and role-play activities, which significantly enhanced their preparedness and confidence in conducting fieldwork.

***B.2. Data collection:***

Eligibility criteria were as follows: age ≥ 18 years; currently residing in India; self-identification as TGD (including socio-cultural identities such as hijra, kinnar, aravani, and people identifying as non-binary, gender-fluid, or gender-nonconforming), and/or identification as a person with a disability (physical, sensory, intellectual, psychosocial, and multiple disabilities). Data were collected using the interviewer-administered REDcap portal, with reasonable accommodations (screen readers, large print, sign language interpretation upon request, and telephone/WhatsApp options).

We used purposive and network-based snowball recruitment via community-led organizations, disability rights collectives, and allied NGOs/government partners. Recruitment leveraged online platforms (e.g., WhatsApp, Telegram, Instagram, and email) and offline leads. Online data collection provided space to gather data from remote locations, as well as going beyond present networks that were bound by geography. However, it also connected the team to more urban, advocacy-linked participants. The sample size was determined pragmatically to balance representation across communities while ensuring feasibility for psychometric analyses. Before collecting data, an in-depth informed consent process was implemented. Team members/enumerators conducted in-depth calls with all the participants, where they described the study to them. Consent was recorded through multiple formats, with options such as written audio recordings, typed texts, etc. The reason for having a flexible consent process was to ensure that study participants would not face barriers to providing their consent.

**C. Data analysis and tool validation phase:**

***C.1. Validation of tool:***

We evaluated the data for internal consistency and validity. We conducted factor analysis to understand emerging factors connected to COVID-19 vaccine uptake within the communities. The aim was to understand the emerging factors that were key to vaccine-related decisions that were specific to the marginalized communities. EFA was conducted to understand the factors. However, due to time and sample size constraints, CFA could not be completed. The data were thoroughly cleaned and analyzed using STATA.

***C.2. Analysis and reporting of community-specific findings:***

In conducting the data analysis, we began with a thorough process of data-cleaning to ensure the accuracy and consistency of responses. This involved identifying and removing missing or inconsistent data points, as well as removing outliers and extreme points. Once the data were cleaned and structured, we proceeded to analyze the dataset through descriptive analysis, polychoric correlations, and exploratory factor analysis (EFA). To enhance criterion validity, specific items were incorporated during the development phase to evaluate concurrent validity, focusing on measures such as time taken to become vaccinated after the vaccine was made available to the specific community, intention to receive a vaccine, and ease of vaccination. Although this was a pilot survey that was originally meant to test and validate the adapted survey tool, the team decided to analyze the survey findings to report the descriptive results. This is because this is a survey conducted with two marginalized communities, and it presents results from a topic that has a dearth of evidence.

### 3.2. About Research Participants

The sample consisted of a diverse group of participants categorized by gender, education level, caste, and employment status (Table 1). It included men, women, trans men trans women, and non-binary people, reflecting a broad spectrum of gender identities. Educational attainment (literacy less than grade five (8), primary (3), up to eighth class (4), secondary (19), higher secondary (up to 12th grade) (61), graduate or higher (61), other (116)) varied widely, with many participants achieving at least a graduate degree, showcasing the educational diversity within the sample. Caste/tribal representation encompassed individuals from Scheduled Castes (48), Scheduled Tribes (6), and Other Backward Classes (67), with a significant number identifying as privileged castes (99). The employment (employed (57), unemployed (141), employed for some parts (22)) status of the sample was primarily characterized by unemployment, indicating potential socioeconomic challenges faced by the participants. Additionally, during the co-development stage, the survey also included items for the intersex community. The research team wanted to hear from intersex people and made sure to include them when building and testing their survey. We talked with intersex advocates and even added questions just for their health needs. But in the end, due to the experiences of stigma, lack of dedicated support networks, and worries about privacy, not enough intersex individuals joined the study. This made it impossible to properly analyze or report on their specific vaccine experiences. As a result, the analysis of items specific to the intersex community could not be carried forward.

## 4. Results

The BeSD adapted survey underwent an intensive validation process, during which the following psychometric properties were seen:

- Internal consistency

Cronbach’s alpha was 0.85 and 0.84 for the tool for TGD and disability community, respectively, showing that the internal consistency of the tool is high.

- Criterion validity

The tool demonstrated good criterion validity for the TGD community. Ease of access was strongly linked to the perception of safety regarding the COVID-19 vaccine (0.47, *p* < 0.05), suggesting that as the ease of access to vaccination increases, so does the perception of safety about the COVID-19 vaccine. Additionally, items like service quality (e.g., medical support at 0.4) and knowing where to be vaccinated (0.51) were positively correlated to better access.

The tool showed good criterion validity for the disability community too. Positive correlations, such as that between ease of access and intention to be vaccinated (0.43), show that better access leads to higher intention to be vaccinated. On the other hand, barriers like a lack of identity cards and poor transport negatively impact the ease of access to vaccination. Service satisfaction was also strongly connected to the ease of access (0.57), showing that better services improve access. Overall, the tool effectively measures factors that affect vaccination behavior.

Secondly, with the intention to understand the vaccination landscape and experiences of TGD and disability communities, a percent analysis was performed. Overall, it was seen that communities were motivated to be vaccinated for several reasons, such as better access to some government services, the ability to use public spaces, a sense of responsibility to others, fear of contracting complex health issues, etc. However, they had to face a series of barriers that limited their access to vaccines as well as affecting their vaccine acceptance. The following (Table 2) is a detailed description of what the survey indicated.

**1.**  **Vaccination Willingness and Confidence**

Across the transgender and gender-diverse (TGD) community, willingness to be vaccinated was high. Seventy percent wanted the COVID-19 vaccine immediately when it was first available, and over 81.5% received it within six months of rollout. This challenges the notion that TGD communities were hesitant about the vaccination. Confidence in vaccine safety reflects a more layered story. While nearly three-quarters (72%) felt moderately or highly confident in the vaccine’s safety overall, this confidence dropped sharply when linked to gender-affirming care. Only 41% felt confident that vaccination was safe in relation to hormone therapy or other gender-affirming treatments. This reflects a unique concern within the TGD community: fear that the vaccine might interfere with vital ongoing treatments like HRT, gender-affirming surgeries, or antiretroviral therapy. One participant captured this anxiety, mentioning they were uncertain about whether it was safe to be vaccinated during or shortly after their surgery. The lack of official guidance left many relying on peers’ experiences or trusted community doctors instead of clear information from health systems.

**2.**  **Stigma and Trust**

Past experiences of stigma strongly shaped engagement with health systems. In our survey, 55% of TGD participants had faced stigma in healthcare settings earlier, and nearly 77% of them expected it to happen again. These experiences echo long-standing patterns of exclusion in healthcare spaces, including disrespectful staff behavior and non-inclusive facilities. ‘Trust’ followed this same pattern. Among TGD respondents, 57% said they trusted healthcare workers “not at all” or only “a little,” and mistrust was toward the health system itself, with 48% reporting low or no trust. This shows that while systems could sometimes earn trust, personal mistrust remained the bigger challenge. For people with disabilities, trust levels were more varied. About 68% expressed moderate to high trust in health systems and nearly 78% in healthcare workers, yet a sizable proportion remained cautious. This uneven trust landscape points to deep divisions in how communities experience the healthcare system.

**3.**  **Motivation to be vaccinated**

Despite mistrust and stigma, the motivation to be vaccinated was strong. Over 68% of respondents recognized its importance for their own health, as well as public well-being and community safety, highlighting a collective sense of responsibility. At the same time, it showed that both extrinsic and intrinsic motivators shaped decisions. This dual motivation of wanting to protect others while also safeguarding oneself suggests that uptake was impeded not by hesitancy but by systemic barriers. Communities were ready and willing to be vaccinated; health systems simply did not always match that readiness.

Participants consistently emphasized that their decisions to be vaccinated were shaped not only by personal beliefs but also by powerful social influences. Among transgender respondents, community norms played a decisive role. Several participants (79%) described directions from dera (a communal residence or household led by a guru that functions as a chosen family and a foundational social institution for many people from the transgender community) heads or respected community leaders as being followed without question, reflecting the authority such figures hold in decision-making. Similarly, advice from trusted doctors or local health providers, like ASHA (community health workers) (53%), was often described as non-negotiable in the disability community. Workplace requirements emerged as another major motivator. Respondents working in both the formal and informal sectors recounted how vaccination certificates were needed to resume jobs, access clients, or even enter workplaces. A total of 94% of disability community respondents noted that workplaces and training centers often required vaccines as a precondition for participation, further reinforcing uptake. Similarly, 92% respondents said access to public spaces such as educational institutions, public transport such as trains and buses, or community events was frequently conditional on vaccination certificates, reinforcing vaccination as a social prerequisite.

Family norms and expectations also strongly influenced participants’ decisions. A total of 83% transgender individuals reported that the opinions of natal/chosen families or close relatives mattered, even when families were not always supportive in other aspects of life. For 92% of respondents with disabilities, family members and caregivers often had dual roles as advisors and as practical facilitators of access. Decisions were rarely made independently; caregivers’ concerns about health and safety directly shaped whether, when, and how people with a disability were vaccinated. In many cases, caregivers also physically accompanied participants to vaccination sites, making their influence both emotional and logistical.

**4.**  **Information on interaction with GAS and chronic diseases**

Safety-related concerns were prominent among participants, particularly in relation to ongoing medical treatments. A total of 43% of transgender and gender-diverse respondents frequently expressed uncertainty about how COVID-19 vaccines might interact with gender-affirming services (GAS), including hormone replacement therapy and recent surgical procedures. Some noted the absence of official guidance tailored to their health needs. The lack of clear, community-specific information often led to hesitation, even among those otherwise motivated to be vaccinated.

For 7% of people with disabilities and others managing chronic conditions such as diabetes, hypertension, or mobility-related disorders, there were concerns centered on whether vaccination might exacerbate existing health issues. Some participants recounted experiences being shared by peers about the side effects of vaccination, like fever or fatigue, being more severe, given their underlying conditions, which reinforced apprehension among those who had not yet been vaccinated.

In practice, this meant many people turned to peers, family, or sympathetic doctors, rather than official sources. While community support networks played a critical role, the lack of official, accessible guidance created uncertainty. Participants emphasized that better information, especially around compatibility with ongoing treatments, would have boosted confidence and satisfaction with vaccination.

**5.**  **Accessibility and Barriers**

For TGD participants, practical challenges defined their vaccination experience. Long waits were a major frustration for people with disabilities, with 40% saying they were too prolonged, and 69% respondents reported that restrooms at vaccination centers were not gender inclusive, which directly affected their comfort. More than half (52%) lacked valid ID cards: a requirement that restricted or delayed their access to vaccines.

Everyday realities added pressure: 54% of the respondents who reported as being employed full time or part time said it was difficult to leave work duties to attend vaccination appointments, while 72% felt staff spent too little time with them. Another 70% described medical support as inadequate, which eroded their confidence in the process. Similarly, for people with disabilities, mobility and affordability created additional hurdles. Over half said that not having an escort made it difficult to access vaccination, underscoring the importance of support mechanisms. Cost was also a concern: 34% reported that affording the vaccine was “not at all easy.” Information barriers reinforced these struggles, as many reported difficulties with accessing reliable details about vaccine safety in relation to their disability.

## 5. Discussion

The present study sought to adapt and validate the WHO Behavioural and Social Drivers (BeSD) survey tool for use among TGD, disability, and intersex (TGDI) communities. Participants expressed uncertainty about the interaction between their health needs, including drugs they might be using and the COVID-19 vaccines, mirroring global findings among people living with HIV. Further, we wanted to understand vaccination experiences among these communities, including confidence, motivation, social influences, and access-related barriers, using the BeSD survey. The survey results also showed that barriers extended beyond individual hesitancy and were instead rooted in systemic inequities across digital platforms, physical environments, and interpersonal interactions. For TGD participants, documentation mismatches, binary-gendered spaces, and fears of discrimination exemplified institutional transphobia. People with disabilities encountered challenges in transportation, caregiver support, and physical accessibility. Both groups showed strong motivation and willingness to be vaccinated. What stood in the way were not attitudes alone, but the systems around them.

Our study reiterated that for TGD communities, stigma and mistrust, and practical barriers, such as long waiting times, non-inclusive facilities, and ID requirements made it harder to become vaccinated [3,7,20,27,28]. Vaccine anxieties emerged from a lack of clear information about vaccine safety in relation to hormone therapy and other gender-affirming care [14]. However, when centers offered inclusive spaces, trained staff, and adequate medical support, participants reported smoother and more satisfying experiences. For the disability community, more than half struggled without escorts, a third found the vaccine unaffordable, and many lacked access to reliable safety information. The literature also highlights the crucial role of accessibility [29], and the need for tailored information on specific health needs [9] for people with disabilities. At the same time, strong staff training, supportive medical care, and manageable waiting times acted as powerful enablers [30]. Importantly, for people with a disability, motivation to be vaccinated included both collective responsibility and safeguarding personal health.

The study highlights how the decision to be vaccinated is strongly embedded in social networks and relational influences. Community leaders, workplace mandates, and family or caregiver expectations all shaped motivations to be vaccinated. These results echo earlier work in India, showing that people from both communities have dynamic interactions with community members, community-based organizations (CBOs), doctors, family members, and caregivers in order to make a decision about COVID-19 vaccination [10]. Globally, community authority figures and employers have been recognized as crucial drivers of uptake in marginalized groups, particularly where trust in state institutions is limited [31,32,33].

Safety concerns also played a central role in shaping vaccine attitudes. Many transgender respondents questioned how vaccines might interact with gender-affirming services (GAS) such as hormone therapy [7,14]. Similarly, people with HIV who were receiving antiretroviral therapy had doubts about the impact of the vaccine on their health [3]. For people with disabilities and others with chronic illnesses, concerns about aggravating existing conditions were common, echoing international research showing that chronic disease patients frequently worried about post-vaccine side effects [3,9,10].

These findings strengthen qualitative findings from a prior study undertaken by the research team that documented structural inequities in vaccine access [7,10]. Together, these results highlight that improving vaccination uptake is also about changing systems. When services are inclusive, respectful, and accessible; when bathrooms are safe, IDs are not an obstacle, staff are trained, and support is readily available, communities engage with greater safety, freedom, and dignity. The path forward is clear: reduce stigma, strengthen trust, and design vaccination programs that truly meet the needs of those most marginalized by health systems.

The analysis underscores that inequities are systemic, rather than episodic. Barriers faced during COVID-19 vaccination reflect broader failures in inclusive healthcare design. Routine immunization programs risk replicating these exclusions unless structural reforms are institutionalized. Intersectionality is key: participants negotiated overlapping stigmas related to gender, disability, caste, and poverty. A transgender woman with a mobility impairment, for example, confronted inaccessible sites alongside transphobic staff attitudes, highlighting compounded vulnerabilities. Strategies to navigate included attending vaccination drives as a group to buffer discrimination, or presenting as cisgender to avoid scrutiny. These strategies highlight resilience, but also the burden of inequitable systems. Ultimately, vaccine access for gender-diverse people and people with a disability depends not only on policy design but also on its faithful implementation. Institutionalizing gender-affirming, disability-inclusive communication, ensuring digital accessibility, and embedding community partnerships are essential steps forward.

## 6. Strengths

A key strength of this study lies in its community-based participatory research (CBPR) design, which ensured that transgender, gender-diverse, and disability communities were not only participants but also active contributors in shaping the research process. Community advisory boards and cognitive interviews guided the adaptation of the WHO BeSD tool, improving both cultural validity and relevance for the Indian context. This level of engagement enhanced trust, increased participation, and allowed for the inclusion of perspectives that are often overlooked in mainstream public health studies.

Another strength is the integration of mixed methods and psychometric validation. The use of exploratory factor analysis with polychoric correlations, alongside reliability testing, strengthened the robustness of the adapted tool. Coupling this with reflexive thematic analysis of qualitative narratives allowed us to capture both the measurable behavioral and social drivers of vaccination and the lived realities of systemic exclusion. This mixed-methods approach provides nuanced evidence that is particularly valuable for informing policy and practice.

## 7. Limitations

At the same time, the project also has a few limitations. Firstly, as recruitment occurred through community networks and organizations, potentially overrepresenting individuals more connected to advocacy groups or urban centers, there is a possibility of sampling bias. Despite the team’s efforts to reach remote areas, they remain underrepresented, which challenges the generalizability of findings to all contexts within India. The project embodied a cross-sectional design; although it was able to capture experiences and perceptions at a single time point, it was not able to report on the ability to examine changes in vaccine uptake or attitudes over time. Also, as snowball sampling was used, keeping CBOs at the center of data collection, community members that might not have had access to the networks of the organizations could not be reached, which limits the generalizability of the tool. Recruiting intersex participants in India was quite challenging, as many face stigma, do not have dedicated support networks, and worry about privacy. Hence, standard outreach rarely works for this group. So, the survey could only focus on transgender people and people with disabilities.

The researchers suggest that future work needs to build more trust and support: ideally by working closely with intersex-led groups to make sure their voices are included. This hesitancy is often grounded in past negative experiences with the medical system, such as lack of informed consent, forced or non-consensual medical interventions during childhood, and erasure or invisibility in health policies. When health campaigns do not offer clear information about how vaccines might affect those with intersex traits, uncertainty and skepticism can increase. This gap is a reminder that most public health research and programs leave out intersex communities, not out of neglect, but because structural and social barriers make their participation much harder. More direct engagement and resourcing are needed for true inclusion. Lastly, as the data were collected through self-report, there is a possibility of recall or social desirability biases, particularly in sensitive domains, such as mistrust of health systems or experiences of stigma.

## 8. Conclusions

This study adapted the WHO BeSD framework to the Indian context, adapting it so that it reflected the realities of transgender and disability communities. By integrating questions that emerged from a range of data resources such as stigma, trust, gender-affirming care, and accessibility, the adapted tool was able to capture barriers and enablers that standard models often fail to capture. This demonstrates how tailoring global frameworks to specific settings and communities can generate more relevant insights and guide inclusive vaccination programs.

Secondly, the study shows that there was willingness to be vaccinated during the COVID-19 pandemic among the transgender, gender-diverse, intersex, and disability communities in India. What limited uptake were not attitudes, but systemic barriers rooted in stigma, exclusionary digital platforms, inaccessible health facilities, and inadequate communication. Participants were motivated by a sense of personal health protection and responsibility toward families and communities, yet their access was constrained by documentation mismatches, long waiting times, physical inaccessibility, and mistrust born of repeated discrimination. Due to stigma, lack of support structures, and confidentiality-related concerns, along with past negative and intrusive experiences with the medical system, recruiting intersex participants in health research has proved to be extremely difficult. This particular limitation further underlines the exclusion of intersex individuals from public health programs due to structural and social challenges and not neglect. Future research should engage directly with intersex-led groups, build trust, and provide resources that address their lived experiences and needs.

These findings highlight that the challenge ahead is not to convince communities of the value of vaccines, but to reform systems so that they are truly inclusive. Ensuring gender-affirming and disability-sensitive healthcare environments, providing clear information tailored to the needs of people undergoing gender-affirming services or managing chronic conditions, and embedding digital and physical accessibility into vaccination delivery are essential steps. Partnerships with community-based organizations must be strengthened to rebuild trust and ensure accountability. By making these structural reforms, future immunization efforts for COVID-19 and other conditions, such as TB, HPV, HIV, and emerging pandemics, can achieve equitable coverage and avoid repeating the exclusions that marked the COVID-19 response.

**Policy Recommendations**

These findings show high initial willingness to be vaccinated, but concerns remain regarding vaccine safety, especially for those undergoing gender-affirming treatments or managing chronic health conditions. Mistrust in the broader healthcare system, fueled by prior experiences of stigma, further hampers vaccine uptake. Based on these insights, we recommend the following:

1. Policymakers should adopt this validated survey to assess and address vaccination barriers that are specific to transgender, gender-diverse, intersex, and disabled individuals. This will help tailor interventions around key concerns, such as safety related to gender-affirming care and chronic health conditions.
2. Respondents repeatedly shared experiences of stigma and a lack of affirmative language. Thus, efforts to improve vaccine access should be part of broader health system reform, addressing anticipated stigma and mistrust. Policymakers must create inclusive healthcare environments to build confidence in marginalized communities.
3. The survey tool can be further adapted for a range of marginalized communities, such as Dalit, Adivasi, and broader LGBTQIA+ communities, to better understand their unique healthcare challenges and increase equity in vaccine and healthcare access.
4. As shared in the context of intersex communities, governments and healthcare providers should work closely with CBOs and NGOs in order to understand lived realities and challenges in the field, with the aim of rebuilding trust in the healthcare system. This can be key to addressing community-specific concerns, such as accessible washrooms or mobile applications, and facilitate community engagement, mobilization, and vaccine uptake.
5. As newer adult vaccines for pandemics and diseases like TB, HPV, mPOX, and HIV are introduced, efforts must ensure safety for marginalized communities. It would be impertinent to understand their unique health needs, such as other medications, affirmative surgeries, supplements and the effects of vaccines on them.
6. Interministerial coordination as both health and disability are State subjects and at the central government level, transgender, intersex, and disability communities are protected by law; law enforcement is the responsibility of the Ministry of Social Justice and Empowerment.

By acting on these recommendations, health systems can improve vaccine access, reduce stigma, and foster trust in healthcare, creating more equitable outcomes for marginalized communities.

## 9. Reflexivity and Positionality

The study team was composed of TGD people and people with disabilities, as well as allies working in disability rights and gender justice. Community partners (CLG and ADB board) that came from the community had lived experience and experience in advocacy, and shaped research questions, instrument adaptation, recruitment, and data interpretation.

## Figures and Tables

**Table 1:** Demographic characteristics of participants (N = 220).

Characteristic	Category	Disability Community (n_1_ = 90)		Transgender Community (n_2_ = 130)	
		Number of Participants (n_1_)	Percentage (%)	Number of Participants (n_2_)	Percentage (%)
**Gender Identity**	Men	54	60.00	63	48.46
	Women	33	36.67	39	30.00
	Non-binary	3	3.33	28	21.54
**Gender Diversity (Disability Community)**	Transmen	-	-	33	25.38
	Transwomen	-	-	65	50
	Non-binary	-	-	21	16.15
	Agender	-	-	4	3.08
	Genderqueer/Genderfluid	-	-	4	3.08
	Hijra/Kinnar/Aravani/Jogappa	-	-	13	10
	Prefer not to say	-	-	6	4.62
	Other	-	-	1	0.77
**Disability Type**	Locomotor disability	53	58.89	-	-
	Blindness	14	15.56	-	-
	Low vision	4	4.44	-	-
	Mental illness	4	4.44	-	-
	Cerebral palsy	3	3.33	-	-
	Others	12	13.34	-	-
**Caste**	Open	20	22.22	28	21.54
	Scheduled Castes (SC)	1	1.11	5	3.85
	Scheduled Tribes (ST)	29	32.22	38	29.23
	Other Backward Classes (OBC)	40	44.44	59	45.38
**Education**	Illiterate	2	2.22	4	3.08
	Literate but less than class 5	1	1.11	2	1.54
	Primary (up to 5th class)	1	1.11	3	2.31
	Middle (up to 8th class)	5	5.56	4	3.08
	Secondary (up to 10th class)	7	7.78	12	9.23
	Higher Secondary (up to 12th class)	15	16.67	46	35.38
	Graduate or Higher	59	65.56	57	43.85
	Any other	-	-	2	1.54
**Employment Status**	Employed	35	38.89	22	16.92
	Unemployed	45	50.00	96	73.85
	Partly employed	10	11.11	12	9.23

**Table 2:** Summary of descriptive statistics from the survey.

Factor	Number of Participants	Percentage
**Attitude towards vaccination (Transgender community)**		
Desire to be vaccinated	91	70%
Vaccinated within 6 months	106	81.5%
Had confidence in safety of vaccine	93	71.5%
Had confidence in compatibility of vaccines with their gender-affirming care and other medicines	53	40.7%
**Trust in vaccination (Transgender community)**		
Had prior experiences of stigma	71	54.6%
Anticipated stigma in COVID-19 vaccination space	77	59.3%
Had trust in healthcare system	62	47.6%
Had trust in healthcare workers	74	56.9%
**Trust in vaccination (Disability community)**		
Had trust in healthcare system	62	68.8%
Had trust in healthcare workers	70	77.7%
**Motivations to be vaccinated (Transgender community)**		
Role of family norms	108	83%
Role of dera leader	103	79.2%
**Motivations to be vaccinated (Disability community)**		
Was vaccinated to access public spaces	83	92.2%
Belief in vaccine being good for health	61	67.7%
Workplace mandates	85	94.4%
Role of family norms	83	92.2%
Recommendation of HCWs	48	53.3%
**Information and support needs (Disability community)**		
Had low accessibility to information about vaccines	45	50%
Had accessibility to vaccination services	41	45.5%
**Information and support needs (Disability community)**		
Had low accessibility to information interaction of vaccines with GAS	56	43%
**Barriers to accessibility (Disability community)**		
Long wait times	36	40%
Lack of medical support	63	70%
Low affordability	31	34.4%
**Barriers to accessibility (Transgender community)**		
Lack of IDs	67	51.5%
Lack of gender inclusive bathrooms	90	69.2%
Staff spent inadequate time	65	72.2
Unable to leave work	70	53.8%

## Data Availability

The data that support the findings of this study will be made available on request. Requests to access data can be directed to the iHEAR VaccinEquity team at ihear@sangath.in. To protect the confidentiality of participants from the transgender and disability communities, de-identified datasets will be shared under controlled access agreements if the participants are comfortable with it.

## Supplementary Materials

The adapted BeSD survey for TGDI people and people with disabilities can be found here: https://www.mdpi.com/article/10.3390/vaccines13111095/s1, Table S1: IHVE2 English Survey.
