# Supplementary material for: Adapting the WHO BeSD COVID-19 Survey to Examine Behavioral and Social Drivers of Vaccine Uptake Among Transgender, Intersex, and Disability Communities in India
Source: Vaccines (Basel). 2025 Oct 24;13(11):1095. doi: 10.3390/vaccines13111095 (PMC12656831; doi:10.3390/vaccines13111095)
Supplement: Supplementary file 1 [file vaccines-13-01095-s001.zip › IHVE2 English Survey (1).pdf]

S.No.	Construct	Question	Pipeline Question (Will open up if people click on certain responses)
**SOCIO DEMOGRAPHIC FORM**			
1	Participant ID	Participant ID: ______________________________	
2	Location	What state did you reside in when you got vaccinated?	
		Name of District _________	
3	Area	Was this area:<br>URBAN<br>RURAL	
4	Age	How old are you currently<br>______ Years	
5	Disability	Are you a person with disability?<br>YES<br>NO<br>NOT SURE	
			(If they choose YES)<br>Which disability do you have?<br>Blindness<br>Low-vision<br>Leprosy Cured persons<br>Hearing Impairment (deaf and hard of hearing)<br>Locomotor Disability<br>Dwarfism<br>Intellectual Disability<br>Mental Illness<br>Autism Spectrum Disorder<br>Cerebral Palsy<br>Muscular Dystrophy<br>Chronic Neurological conditions<br>Specific Learning Disabilities (Dyspraxia, Dysgraphia, Dyscalculia, Attention Deficit and Hyperactivity Disorder (ADHD))<br>Multiple Sclerosis<br>Speech and Language disability<br>Thalassemia<br>Hemophilia<br>Sickle Cell disease<br>Multiple Disabilities including deaf blindness<br>Acid Attack victim<br>Parkinson's disease<br>Any other disability not on this list___________________________
6	Gender	What is your gender?<br>WOMAN<br>MAN<br>NON-BINARY PERSON	
7	Transgender identity	Are you a transgender person?<br>YES<br>NO<br>NOT SURE	(If they choose YES)<br>Would you say you describe yourself as a:<br>TRANS MAN OR TRANS MASCULINE PERSON<br>TRANS WOMAN OR TRANS FEMININE PERSON<br>NON-BINARY PERSON<br>AGENDER PERSON<br>NON-BINARY PERSON<br>GENDER QUEER, GENDER FLUID PERSON<br>GENDER NON-CONFORMING PERSON<br>HIJRA, KINNAR, ARAVANI, SHIVASHAKTIS, JOGAPPA<br>PREFER NOT TO SAY<br>ANY OTHER TRANS* IDENTITY NOT MENTIONED HERE<br>___________________
7	Intersex variation	Are you a person with intersex variation?<br>YES<br>NO<br>NOT SURE<br>I DO NOT KNOW WHAT THIS QUESTION IS ASKING	
8	Education	What is your highest level of education?<br>NON-LITERATE<br>LITERATE BUT BELOW PRIMARY<br>PRIMARY<br>MIDDLE<br>SECONDARY<br>HIGHER SECONDARY<br>GRADUATE OR HIGHER<br>OTHER __________	

9	Employment status	What was your employment status when the COVID19 vaccination process was ongoing (2020-2023) ?<br>Employed<br>Unemployed<br>Employed for some parts of the process	
			(If the participant answers, Employed or Employed for some parts of the process)<br><br>WHAT WAS THE NATURE OF YOUR EMPLOYMENT<br><br>Formal employment<br>Informal employment<br>Self-employed"
10	Religion	Can you tell me which religious group you belong to?<br>____________________	
11	Caste	Which caste category do you belong to?<br>SCHEDULED CASTE<br>SCHEDULED TRIBES<br>OTHER BACKWARD CLASSES<br>GENERAL/UNRESERVED	
12	Comorbidities and COVID19 risk	Do you have a chronic condition?<br>This could include, for example, obesity, diabetes, lung disease, HIV, hypertension or another long-term condition or it's treatment.<br>NO<br>YES<br>NOT SURE	
13	COVID19 Dignosis	To your knowledge, have you ever had COVID-19?<br>NO<br>YES	
			(If the participant answers, Yes)<br>Was it...<br>Confirmed by a test, or<br>Not confirmed by a test?

MAIN SURVEY QUESTIONS

1	Perceived risk to self	How concerned were you about getting COVID-19, during the pandemic? Would you say...<br>NOT AT ALL CONCERNED,<br>A LITTLE CONCERNED,<br>MODERATELY CONCERNED, OR<br>VERY CONCERNED?	
2	COVID-19 Vaccine Uptake	Have you received a COVID-19 vaccine? Would you say...<br>NO<br>YES, YOU RECEIVED ONE DOSE<br>YES, YOU RECEIVED TWO DOSES, OR<br>YES, YOU RECEIVED THREE OR MORE DOSES?<br>NOT SURE	
			(If the participant answers, Yes, you received one dose, Yes, you received two doses, Yes you received three or more doses)<br>How long did it take you to get your first dose after vaccine was made available to their age group for the first time?"<br>0-3 Months<br>3-6 months<br>6-9 months<br>9 months and more
3	Intention to get vaccinated	Did you want to get the COVID-19 vaccine when it was first made available? Would you say...<br>NO, YOU DID NOT WANT TO<br>YES, YOU WANTED TO<br>YOU ARE NOT SURE	

4	Confidence in COVID-19 vaccine benefits - Health	How important did you think getting a COVID-19 vaccine was for your health? Would you say…<br>NOT AT ALL IMPORTANT,<br>A LITTLE IMPORTANT,<br>MODERATELY IMPORTANT, OR<br>VERY IMPORTANT?	
5	Confidence in COVID-19 vaccine safety - Overall	How safe did you think a COVID-19 vaccine was for you?<br>NOT AT ALL SAFE,<br>A LITTLE SAFE,<br>MODERATELY SAFE, OR<br>VERY SAFE?	
6	Confidence in COVID-19 vaccine safety - Concerns about interaction with chronic conditions and Comorbidities	(Only for people who report chronic conditions in SDQ)<br>How confident were you about the safety of the COVID-19 vaccine with respect to your chronic or long-term health condition(s)? This could include, for example, obesity, diabetes, lung disease, cancer, HIV or another long-term condition or its treatment.<br>NOT AT ALL CONFIDENT<br>A LITTLE CONFIDENT<br>MODERATELY CONFIDENT<br>VERY CONFIDENT	
7	Access to Information on specific health needs - comorbidities and long term conditions		(Only for people who report chronic conditions in SDQ)<br>How easy was it for you to get information on the safety of the COVID-19 vaccine for people with your chronic health conditions like diabetes, HIV, cancer, etc.<br>NOT AT ALL EASY,<br>A LITTLE EASY,<br>MODERATELY EASY, OR<br>VERY EASY?
8	Confidence in COVID-19 vaccine safety - Concerns about interaction with gender-affirming services		(For respondents from the trans* and intersex community)<br><br>How confident were you about the safety of the COVID-19 vaccine for people who have used or are using gender-affirming services? For example, hormone replacement therapy (HRT), gender-affirming surgeries (GAS) also known as sex reassignment surgeries (SRS)<br>NOT AT ALL CONFIDENT<br>A LITTLE CONFIDENT<br>MODERATELY CONFIDENT<br>VERY CONFIDENT
9	Access to Information on specific health needs - Information on interaction with gender-affirming services		(For respondents from the trans* and intersex community)<br>How easy was it to get information on the safety of the COVID-19 vaccine for people who have used or are using gender-affirming services? For example, hormone replacement therapy (HRT), gender affirming surgeries (GAS) also known as sex reassignment surgeries (SRS)?<br>NOT AT ALL EASY,<br>A LITTLE EASY,<br>MODERATELY EASY, OR<br>VERY EASY?
10	Confidence in COVID-19 vaccine safety - Concerns about interaction with disability		(For respondents with disability)<br>How confident were you about the safety of the COVID-19 vaccine for people with your disability?<br>NOT AT ALL CONFIDENT<br>A LITTLE CONFIDENT<br>MODERATELY CONFIDENT<br>VERY CONFIDENT
11	Access to Information on specific health needs - disability community		(For respondents with disability)<br>How easy was it for you to get information on the safety of the COVID-19 vaccine in relation to your disability?<br>NOT AT ALL EASY,<br>A LITTLE EASY,<br>MODERATELY EASY, OR<br>VERY EASY?

12	Access to Information on specific health needs - intersex bodies		(For respondents from the intersex community)<br>How easy was it for you to get information on the safety of the COVID-19 vaccine in relation to intersex variations?<br>NOT AT ALL EASY,<br>A LITTLE EASY,<br>MODERATELY EASY, OR<br>VERY EASY?
13	Trust in health systems	How much did you trust the systems like the government and health systems that were involved in making and providing the COVID-19 vaccination? Would you say…<br>NOT AT ALL,<br>A LITTLE,<br>MODERATELY, OR<br>VERY MUCH?	
14	Trust in health care worker	How much did you trust the health worker who was going to give you the COVID-19 vaccine?<br>NOT AT ALL,<br>A LITTLE,<br>MODERATELY, OR<br>VERY MUCH?	
15	Anticipated stigma - Prior experiences of stigma	Did your face any negative past experiences within healthcare settings like discrimination or stigma?<br>NO<br>YES	
			(If the participant answers, Yes)<br>Did your negative past experiences within healthcare make you concerned about visiting a COVID-19 vaccination centre?<br>NO<br>YES<br>NOT SURE"
16	Family norms	Did you think most of your close family and friends wanted you to get a COVID-19 vaccine? This could include friends, partners, others in your gharana/dera etc. ?<br>NO<br>YES	
17	Access to public spaces and services	Did you think getting a COVID-19 vaccine was important for accessing public spaces and services like public transport again?<br>NO<br>YES	
18	Workplace mandate	Did you think getting a COVID-19 vaccine was important for accessing your workplace or continuing your job?<br>NO<br>YES<br>I WAS NOT WORKING AT THE TIME	
19	Community peer norms	Do you think other commuity members in trans/intersex and disability community wanted you to get the COVID-19 vaccine?<br>NO<br>YES	
20	Health worker recommendations	Did a health worker like a doctor, ASHA, ANM, NGO worker, recommend you get a COVID-19 vaccine?<br>NO<br>YES	
21	Know where to get vaccination	Did you know where to get the COVID-19 vaccination? Would you say…<br>NO<br>YES	
22	Ease of Access	How easy was it to get a COVID-19 vaccine for yourself? Would you say…<br>NOT AT ALL EASY,<br>A LITTLE EASY,<br>MODERATELY EASY, OR<br>VERY EASY?	

24	Reasons for low ease of access	What made it hard for you to get a COVID-19 vaccine? Would you say…<br><br>[READ ALOUD ALL RESPONSE<br>OPTIONS, PAUSING AFTER EACH<br>TO ALLOW RESPONDENT TO<br>ANSWER “YES” OR “NO” AFTER<br>EACH RESPONSE OPTION.<br>RESPONDENTS MAY SELECT<br>MULTIPLE RESPONSE OPTIONS.]	
		Not having a valid identity card made it hard<br>Yes<br>No	
		The vaccination site was hard to get to,<br>Yes<br>No	
		The opening times were inconvenient,<br>Yes<br>No	
		The waiting time took too long,<br>Yes<br>No	
		I was unable to leave work duties,<br>Yes<br>No	
		I was turned away without vaccination<br>Yes<br>No	
25	Reasons for low ease of access.- Availability of gender concordant ID card		(For trans* participants)<br>What made it hard for you to get a COVID-19 vaccine? Would you say…<br>Having to show an identity card at the center when my identity card does not reflect my gender identity<br>Yes<br>No
26	Reasons for low access - accessibility of information		(For persons with disability) What made it hard for you to get a COVID-19 vaccine? Would you say…
			It was hard to find information on COVID-19 vaccination in accessible formats like sign language interpretation, screen reader-friendly information etc.<br>Yes<br>No
			It was hard to find accessible transport to go to the centre<br>Yes<br>No
			It was hard to reach the centre without a caregiver or an escort.<br>Yes<br>No
27	Service satisfaction	How satisfied were you with COVID-19 vaccination services? Would you say…<br>NOT AT ALL SATISFIED,<br>A LITTLE SATISFIED,<br>MODERATELY SATISFIED, OR<br>VERY SATISFIED?	

28	Service quality	What was not satisfactory about the COVID-19 vaccination services? Would you say… <br>[READ ALOUD ALL RESPONSE OPTIONS, PAUSING AFTER EACH TO ALLOW RESPONDENT TO ANSWER “YES” OR “NO” AFTER EACH RESPONSE OPTION. RESPONDENTS MAY SELECT MULTIPLE RESPONSE OPTIONS.]	
		Vaccine was not available at the center,<br>Yes<br>No	
		There were delays in the opening time of vaccination site.<br>Yes<br>No	
		Waiting times were long,<br>Yes<br>No	
		Vaccination site was not clean,<br>Yes<br>No	
		Staff were poorly trained,<br>Yes<br>No	
		Staff were not respectful,<br>Yes<br>No	
		Staff did not spend enough time with people,<br>Yes<br>No	
		Medical support (post vaccination support, counseling) at the centre was not adequate or<br>Yes<br>No	
		Is there something else? ________	
29	Service quality - Trans community		(For trans* respondents)<br>What was not satisfactory about the COVID-19 vaccination services? Would you say…<br><br>[READ ALOUD ALL RESPONSE OPTIONS, PAUSING AFTER EACH TO ALLOW RESPONDENT TO ANSWER “YES” OR “NO” AFTER EACH RESPONSE OPTION.RESPONDENTS MAY SELECT MULTIPLE RESPONSE OPTIONS.]
			The gender categories like male, female, other etc. collected during COVID-19 vaccination registration were not inclusive.<br>Yes<br>No
			Bathrooms at the centre were not transgender inclusive<br>Yes<br>No

30	Service quality - Accessibility of services		(For respondents with disability) <br>Persons with disability often have to face a variety of unique challenges while moving around hospitals and clinics due to social and infrastructural barriers. This can include lack of ramps, accessible transport, easy-to-read signages or overstimulating sensory environments and much more. In your experience, how accessible were the COVID-19 vaccination services? NOT AT ALL ACCESSIBLE, A LITTLE ACCESSIBLE, MODERATELY ACCESSIBLE, OR VERY ACCESSIBLE?
			(If the participant answers, Yes)<br><br>COULD YOU SHARE WHAT KIND OF ACCESSIBILITY CHALLNEGES DID YOU FACE WHILE TRYING TO GET THE COVID 19 VACCINATION?<br>______________________________
31	Service quality - Access to Home Vaccinations		(For respondents with disability) Did you need a home vaccination? NO YES
			(If the participant answers, Yes)<br><br>How easy was it to recieve a home vaccination? NOT AT ALL EASY, A LITTLE EASY, MODERATELY EASY, OR VERY EASY?
